# Institutional Needs

**Published:** 1913-10-04

**Authors:** 


### INSTITUTIONAL NEEDS.

### NEW AWARD FOR "RONUK."

AT the exhibition in connection with the seventeenth meeting of the International Congress of Medicine, the Ronuk Company was awarded a gold medal for their sanitary polishes and appliances.

### THE GAS-STOVE FOR INSTITUTIONS.

THE "Altheat" System of Heating is a method by which coal or petrol-air gas is used to warm the air of a room, without making it arid to breathe. The "Altheat" combined stove and radiator is based upon the two simple laws that vapour suddenly condensed produces a vacuum, and that when two vapours of different temperatures meet condensation quickly occurs. In the stove, therefore, the hot fumes are caused to enter a receiver, where they lose their heat by convection. They then pass through dense vapour, and re-enter the flue pipe into which they were first discharged, where they meet the hot fumes entering the receiver. Condensation thus occurs, a vacuum is created, and thus more fumes are made to enter the receiver; and the circulation above described becomes continuous. Moreover, the required draught is produced without unduly encroaching on the air of the room. This ingenious and scientific contrivance, which has been constructed by the Coalbrookdale Co., Ltd., is designed to replace a fire-grate, and has successfully avoided the hideousness of the older type of gas-stove. It is stated that, in addition to the radiant heat given off by the incandescent surface of the stove, the radiator gives off heat at 105° F. from 2,300 sq. in. of surface. The consumption of gas to heat a room, say, 20 feet square, is estimated at 10 cubic feet only. A quarter-inch gas pipe is sufficient, and already institutions are trying the stove. One which was examined a little while ago was stated by its owner to be quite free from smell, to have no back draught, and to be giving continuous satisfaction. The "Altheat" combined stove and radiator can be obtained from the "Altheat" Co., Ltd., Evelyn House, 62 Oxford Street, London, W.

### TWO NOVELTIES FOR DISPENSERS.

THE BAYER Co., 19 St. Dunstan's Hill, E.C., has submitted to us two novelties which deserve the attention of institutional medical officers and dispensers. One is hydrastinine hydrochloride in tablet form, silver coated. This is prepared synthetically, and has proved to be in every way identical with the alkaloid obtainable from the rhizome of the hydrastis plant by oxidation of the hydrastine which the latter contains. Hydrastinine has such well-marked superiority over hydrastine that its synthesis and the absolute freedom from contamination which this method of preparation involves constitute a noteworthy advance in pharmacology. The other new preparation is named "Tablet Helmitol Compound," and consists of a mixture of helmitol and acid sodium phosphate in equal parts. The addition of the phosphate has been found valuable in dealing with conditions of pronounced urinary alkalinity, and it will undoubtedly prove a convenience to prescribers to have the combination procurable ready made.

### THE HOSPITAL OFFICER'S TONIC.

NOWADAYS, for prescribers and patients alike, the question is not what tonic shall be taken, but which preparation of established ingredients. "Vitafer," a cream-coloured granulated powder, readily soluble, may be roughly described as a form of concentrated or dried milk with the addition of certain phosphates and magnesium salts, the distinction aimed at by the careful combination of these constituents being a tonic with the richness in food quality of milk, but without milk's tendency to produce constipation. It is, of course, well-known to institutional officials, who, whether run down by the exacting demands of superintendence, with all the anxieties such a position entails, or whether it be a nurse suddenly changed from day to night duty, are all generally liable to strain and consequent debility. "Vitafer" is also designed as a food for diabetic patients, and the Shropshire factory is busily occupied in meeting the demand. It is also only fair to mention that "Vitafer," of which the manufacturers are Messrs. Southall Bros. and Barclay, Ltd., of Birmingham, is a cheaper preparation than are certain well-known rivals which have the disadvantage of being non-British in manufacture.

### SWANSDOWN TOWELETTES.

THE many different varieties of sanitary towels on the market, both of the washable and burnable types, show the difficulty of designing a really satisfactory and absorbent towel, free from compensating disadvantages. Of the washable type the "Mrs. Evaline" Washable Swansdown Towelette has many claims to perfection. Made of four folds of Turkish towelling, these Towelettes possess the advantages of a washable diaper, with the known softness and absorbent qualities that the above substance, when provided with "an inner swansdown surface," is expected to give. In addition to these hygienic qualities they are fitted so as conveniently to be adjusted to any belt or suspender or tape. They may be obtained of all chemists, drapers, and outfitters at prices varying from 4s. to 6s. 6d. per dozen, according to size, or from Mrs. Evaline, Bradford, Yorks.

